# Clinical and Cost Implications of Insulin Degludec in Patients with Type 1 Diabetes and Problematic Hypoglycemia: A Quality Improvement Project

**DOI:** 10.1007/s13300-018-0400-x

**Published:** 2018-03-16

**Authors:** Muhammad Ali Karamat, Shujah Dar, Srikanth Bellary, Abd A. Tahrani

**Affiliations:** 1 Department of Diabetes and Endocrinology, Diabetes Centre, Heartlands Hospital, Birmingham, UK; 2 Institute of Metabolism and Systems, School of Clinical and Experimental Medicine, University of Birmingham, Birmingham, UK; 3 Centre of Endocrinology, Diabetes and Metabolism, Birmingham Health Partners, Birmingham, UK; 4 Department of Diabetes and Endocrinology, Queen Elizabeth Hospital, Birmingham, UK; 5 School of Life and Health Sciences, Aston University, Aston Triangle, Birmingham, UK

**Keywords:** Healthcare delivery, Hypoglycemia, Insulin degludec, Insulin therapy, Type 1 diabetes

## Abstract

**Introduction:**

To assess the real-life clinical benefits and cost implications of switching from another basal insulin to insulin degludec (degludec) in patients with type 1 diabetes (T1D) on basal–bolus regimens with recurrent hypoglycemia and/or hypoglycemia unawareness.

**Methods:**

Patients with T1D who were aged ≥ 18 years, were on a basal–bolus regimen, and had switched to degludec plus bolus insulin for at least 6 months were included. Patients had to have switched to degludec as a result of recurrent hypoglycemia and/or hypoglycemia unawareness.

**Results:**

Six months of follow-up data were available for 42 patients. At 6 months, there was a significant reduction in median (interquartile range) HbA_1c_, from 8.6 (8.0–9.3)% [70 (64–78) mmol/mol] to 8.4 (7.9–8.9)% [68 (63–74) mmol/mol]; *p *< 0.05. Median daily basal insulin dose reduced significantly from 30.0 (14.7–45.0) to 25.5 (14.0–30.2) units; *p *< 0.0001. Data from hospital records showed reductions in the frequency of episodes of severe hypoglycemia from eight in the 6 months preceding degludec initiation to two in the 6 months following initiation. In the same period, diabetic ketoacidosis (DKA) episodes reduced from two before degludec initiation to no episodes after initiation. No patients reported worsening treatment satisfaction after switching to degludec. Considering the reductions in the basal dose required and the frequency of hypoglycemia episodes, we estimate that switching such patients to degludec from other basal insulins could provide significant savings in direct healthcare costs.

**Conclusion:**

In patients with T1D, switching to degludec was associated with an improvement in HbA_1c_ and reductions in basal insulin dose, severe hypoglycemia, and DKA. When used in appropriate patients, degludec could lead to significant cost savings.

**Funding:**

Novo Nordisk.

## Introduction

Hypoglycemia in patients with type 1 diabetes (T1D) represents a considerable economic burden to the UK National Health Service (NHS). Data from the West Midlands Ambulance Service show that between January 1, 2013 and December 31, 2014 in the West Midlands region of the UK (covering a population of 5.6 million [1]), there were 3813 ambulance call-outs related to hypoglycemia, costing the NHS over £900,000 [2]. During the same period, hospital admissions attributed to a primary diagnosis of hypoglycemia were estimated to cost £464,760 [3]. Hypoglycemia can have considerable negative effects on patient quality-of-life (QoL), with an increased frequency and severity of hypoglycemic episodes associated with greater reductions in QoL [4]. Severe hypoglycemia is associated with an increased risk of falls, fractures, cardiovascular disease, major adverse cardiovascular events, major microvascular events, dementia (in older patients) and death [5–8].

Insulin degludec (degludec) is a long-acting basal insulin with a duration of action extending to over 40 h in most patients at therapeutic doses [9]. It has a flat pharmacodynamic profile at steady state, with less injection-to-injection variability in glucose-lowering activity than insulin glargine 100 units/mL (glargine U100) [9, 10]. Randomized controlled trials (RCTs) in patients with T1D and type 2 diabetes (T2D) have shown that, compared with glargine U100, degludec leads to a reduced risk of hypoglycemia, particularly nocturnal hypoglycemia, at equivalent levels of glycemic control across a broad spectrum of patients [11–13]. Degludec has also been associated with improved QoL scores in phase 3 clinical trials compared with glargine U100 in T1D and T2D [14]. RCTs are the gold standard for developing evidence on the safety and efficacy of new treatment options, but their well-known limitations, such as their strictly defined inclusion and exclusion criteria, restrict the generalizability of their results to the more heterogeneous populations of patients encountered in clinical practice [15]. Real-world studies can provide a valuable additional source of evidence that complements clinical trial data and helps to bridge the knowledge gap between RCTs and real-world practice. To date, a small number of non-interventional studies have evaluated the clinical effectiveness and safety of switching to degludec from other basal insulins in real-world clinical practice [16–18]. These studies report improved glycemic control and a reduced risk of hypoglycemia after switching to degludec from regimens involving other basal insulins in patients with T1D or T2D [16–18].

A recent short-term health economic analysis based on data from the phase 3a registration trials and modeling based on the UK NHS setting found degludec to be dominant (more effective at a lower cost) over glargine U100 in patients with T1D or patients with T2D on basal-only therapy, and cost-effective for T2D and a basal–bolus regimen [19]. Scenario analyses versus two recently marketed basal insulin analogs indicated that degludec would likely be cost-effective versus glargine 300 units/mL and biosimilar glargine across diabetes types and treatment regimens [19]. However, the trials included in this analysis excluded patients with a history of recurrent hypoglycemia and those at a high risk of hypoglycemia, such as patients with hypoglycemic unawareness, where degludec might be particularly beneficial in a clinical setting.

This prospective, real-world study was therefore undertaken to investigate the effects of switching basal insulin to degludec in T1D patients with problematic hypoglycemia, including hypoglycemia unawareness. Study endpoints included changes in glycemic control, basal insulin dose, rates of hypoglycemia, and changes in hypoglycemia unawareness status and QoL. An exploratory analysis of the cost-effectiveness of switching to degludec was also carried out.

## Methods

**Fig. 1:**
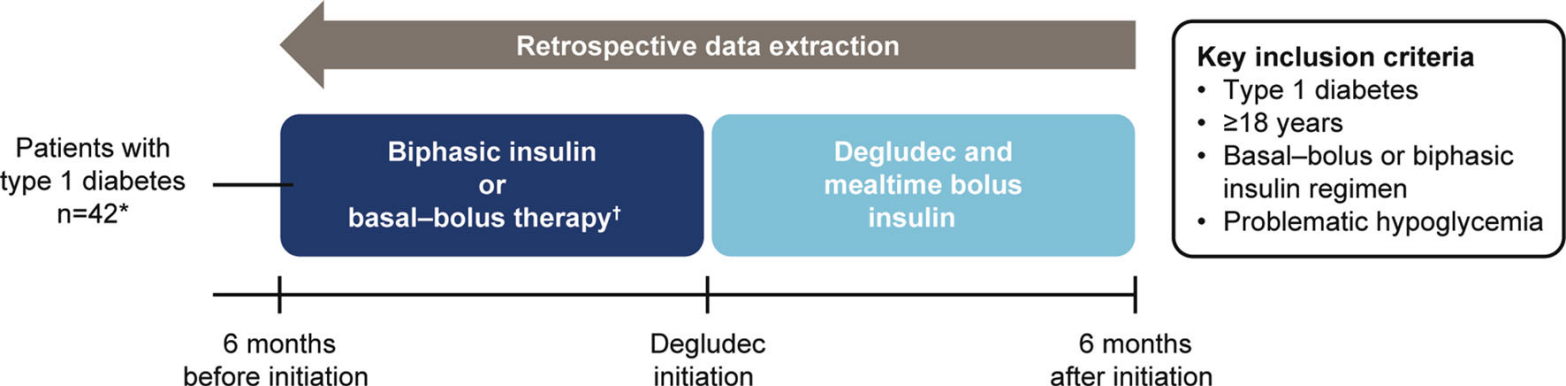
Quality improvement project design. Asterisk indicates a total of 45 patients were included in the project, three of whom discontinued degludec and initiated continuous subcutaneous insulin infusion at the physician’s discretion; Dagger symbol indicates excluding regimens with degludec

This was a prospective, quality-improvement project in patients with T1D attending the outpatient clinics at the Heart of England NHS Foundation Trust (the Trust) (see Fig. 1 for project design). Data were extracted retrospectively to compare clinical outcomes and QoL in patients with T1D and problematic hypoglycemia who were switched to degludec from regimens that included other basal insulins, as part of routine clinical practice. The audit was registered and the data were collected as part of routine clinical care and subject to the Trust’s usual governance regulations. Patients were included in the study if they:

- Had T1D and attended the outpatient clinics at the Trust;
- Were aged ≥ 18 years;
- Were on an insulin regimen comprising either a basal–bolus regimen or biphasic insulin, and
- Had one or more of the following:
  - Recurrent severe or nocturnal hypoglycemia
  - Documented hypoglycemia unawareness, as identified from the patients’ blood glucose (BG) diaries and/or data from a continuous glucose monitor.

All eligible patients were reviewed by a physician and offered the option of switching their basal insulin or biphasic insulin after excluding all other reasonable options to improve control, such as changing the timing of the basal insulin injections and splitting the dose between more than one daily injection. All patients had also received structured diabetes education, such as the Bournemouth Type 1 Diabetes Education Programme, well before commencing treatment with degludec, as part of routine clinical practice for patients with T1D. No education programs were initiated as part of the treatment switch. Eligible patients were then switched to degludec and any previous basal/biphasic insulins were discontinued. Patients were advised to take degludec administered daily in the morning, initiated with a 20% dose reduction from the previous basal insulin dose. Doses were subsequently titrated as required. Bolus insulin was administered at mealtimes. All patients were followed up for a period of 6 months.

Demographic details, HbA_1c_ levels, previous basal insulin dose, previous episodes of diabetic ketoacidosis (DKA), and episodes of hypoglycemia [defined as BG ≤ 70.3 mg/dL (3.9 mmol/L)] were recorded for all patients for 6 months before and for 6 months after the initiation of degludec. Episodes of hypoglycemia were recorded via home BG monitoring or continuous glucose monitoring systems (for a subset of patients) and recorded in patients’ BG diaries. Data on the number of episodes of DKA and ambulance call-outs for 6 months before and 6 months after starting degludec were obtained from hospital records.

At degludec initiation and at the 6-month follow-up, patients completed a diabetes questionnaire based on the Clarke Hypoglycemia Questionnaire as part of routine clinical care for patients with T1D and problematic hypoglycemia [20]. This is a self-reported measure of the number of episodes of hypoglycemia and of the extent that patients experienced hypoglycemia unawareness. At the 6-month follow-up, patients completed a satisfaction questionnaire, which included questions about fear of hypoglycemia, confidence in efficacy of treatment, predictability of BG readings, concerns over diabetes, treatment satisfaction, and whether the patient was happy to continue treatment.

### Statistical Analysis

Paired-sample *t* tests were carried out to compare changes in HbA_1c_ at baseline and at the 6-month follow-up. A related-sample Wilcoxon signed-rank test was used to compare basal doses at baseline (before the switch to degludec) and at the 6-month follow-up. Missing data were dealt with using list-wise deletion.

**Fig. 2:**
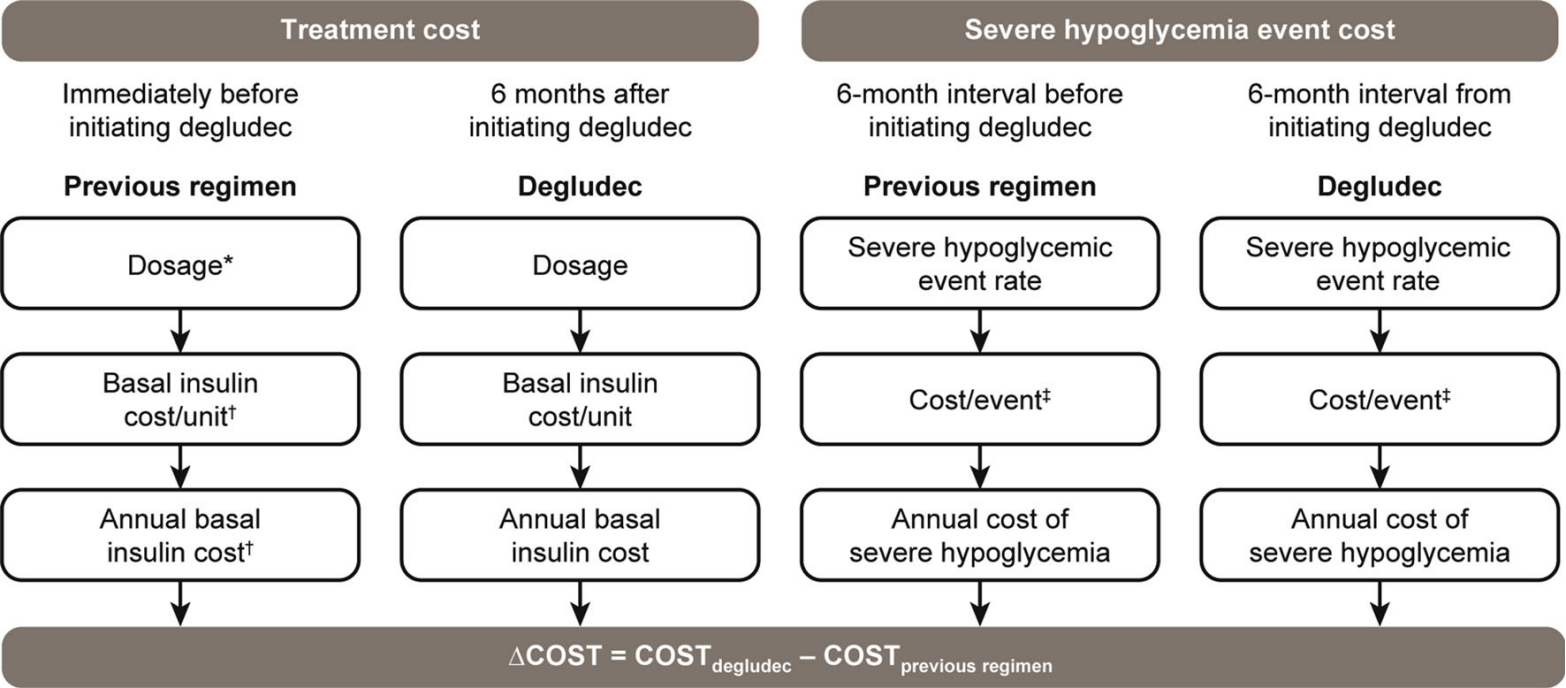
Overview of the cost-effectiveness model for switching to degludec from other basal insulins.* Previous regimens* included basal–bolus therapy and biphasic insulin. Dosage was the mean basal insulin dose.* Severe hypoglycemia* was defined as an event requiring an ambulance call-out for the purposes of this analysis. Asterisk indicates that two patients who received biphasic insulin before initiating degludec were included; dagger symbol indicates that the costs were calculated for glargine 100 units/mL; double dagger symbol indicates the direct cost of an episode (excluding ambulance call-out and/or hospital admission) for Birmingham and the surrounding area

An exploratory cost-effectiveness analysis was performed to provide an illustrative example of the costs associated with switching to degludec from other basal insulins in patients with T1D and problematic hypoglycemia as part of routine clinical practice. For the purposes of this cost analysis, severe hypoglycemia was defined as an episode requiring an ambulance call-out and/or hospital admission. Data from hospital records and the West Midlands Ambulance Service were used to estimate the change in the number of severe hypoglycemic events experienced in the 6-month period before and after switching to degludec. These data and basal insulin costs (taken as glargine U100 for the pre-switch arm) based on UK list prices [21] were used to estimate any cost savings [in pounds sterling (GBP), based on 2016 costs and values] caused by patients switching to degludec (due to reductions in the number of severe hypoglycemic events and the basal insulin dose); see Fig. 2 for an overview of the cost-effectiveness model. The average annual change in cost per patient attributable to the reduced incidence of severe hypoglycemia upon switching to degludec was offset against the difference in the average annual insulin treatment costs, accounting for any change in dose requirement.

### Compliance with Ethics Guidelines

This article is based on previously conducted studies and does not contain any studies with human participants or animals performed by any of the authors.

## Results

### Baseline Characteristics

A total of 45 patients were initially approached to be included in the study, but three patients discontinued degludec and were switched to continuous subcutaneous insulin infusion, at their physician’s discretion. Therefore, 42 patients met the inclusion criteria and provided data. Demographic and clinical characteristics of the cohort are shown in Table 1. Degludec dose values at initiation were missing for four patients; degludec dose values at follow-up were missing for eight patients; HbA_1c_ values at follow-up were missing for four patients. Some of this missing information was the result of failure to attend the 6-month follow-up clinic appointments. At the beginning of the study, the basal insulins given to the patients were glargine U100 (Lantus^®^, Sanofi–Aventis US LLC, Bridgewater, NJ, USA; *n* = 23); insulin detemir (Levemir^®^, Novo Nordisk A/S; *n* = 17); biphasic insulin lispro (Humalog^®^ Mix25^®^, Eli Lilly Nederland B.V., Utrecht, The Netherlands; *n* = 1; Novomix30, *n* = 1); or Insulatard^®^ (Novo Nordisk A/S; *n* = 1). At degludec initiation, 31 (74%) patients were experiencing disabling and/or recurrent nocturnal hypoglycemia and 11 (26%) had hypoglycemia unawareness.

**Table 1:** Baseline characteristics of the patients enrolled in the study

Characteristic	Baseline value
Number of patients	42
Mean age, years (± SD) Range, years	50.4 (± 14.0) 22–80
Female/male (%)	64.3/35.7
Duration of diabetes, years (± SD) Range (years)	26.1 (± 12.2) 4–48
HbA_1c_, % (± SD) [mmol/mol] Range, % [mmol/mol]	8.7 (± 1.3) [72 (14)] 5.9–12.2 [41–110]
Mean basal insulin dose pre-switch (U) Range (U)	36.8 (29.4) 6–150
Mean degludec starting dose (U) Range (U)	29.2 (23.7) 4–120

*HbA*_*1c*_ glycated hemoglobin, *SD* standard deviation, *U* units

### Endpoints

At the 6-month follow-up, there was a significant reduction in mean (standard deviation) HbA_1c_ from 8.7 (1.3) % [72 (14) mmol/mol] to 8.4 (1.1) % [68 (12) mol/mol]; *p* < 0.05. Median (interquartile range) daily basal insulin dose reduced significantly from 30.0 (14.7–45.0 units to 25.5 (14.0–30.2) units; *p* < 0.0001. For the 39 patients previously on another basal–bolus regimen, data on insulin (carbohydrate ratios, type, and average dose of bolus) were available for 31 of them. Of these, only one patient’s insulin:carbohydrate ratio had increased at follow-up; eight reduced and 22 did not change.

Two patients experienced an episode of DKA in the 6 months before degludec initiation; there were no DKA events in the 6 months after degludec initiation. There were eight episodes of severe hypoglycemia in the 6 months before degludec initiation, and two episodes in the 6-month follow-up period.

All patients completed the Clarke Hypoglycemia Questionnaire (Table 2) and the Patient Satisfaction Questionnaire (Table 3). The percentage of patients who reported experiencing more than two episodes of moderate hypoglycemia in the past 6 months decreased from 31% before to 8% after degludec initiation. No patients reported worsening treatment satisfaction after switching to degludec.

**Table 2:** Results from the Clarke Hypoglycemia Questionnaire

Question	Number of patients (%)	
	6 months before degludec initiation	6 months after degludec initiation
How often patients experienced moderate hypoglycemia		
Never	13 (31)	33 (77)
Once or twice	16 (38)	6 (15)
More than twice	13 (31)	3 (8)
Lowest blood glucose range in which symptoms were experienced		
59.5–68.5 mg/dL (3.3–3.8 mmol/L)	17 (41)	25 (59)
50.5–57.7 mg/dL (2.8–3.2 mmol/L)	11 (25)	11 (25)
< 50.5 mg/dL (< 2.8 mmol/L)	14 (34)	6 (16)
Extent that hypoglycemia could be predicted by symptoms		
Rarely	20 (46)	3 (8)
Sometimes	6 (15)	6 (15)
Often	3 (8)	10 (23)
Always	13 (31)	23 (54)

**Table 3:** Results from the Patient Satisfaction Questionnaire

Question	Number of patients (percentage) 6 months after degludec initiation
Fear of hypoglycemia	
Worse	0 (0)
No change	9 (21)
Improved	26 (62)
Much improved	7 (17)
Confidence in efficacy of treatment	
Worse	0 (0)
No change	3 (7)
Improved	38 (91)
Much improved	1 (2)
Predictability of blood glucose readings	
Worse	0 (0)
No change	6 (14)
Improved	34 (81)
Much improved	2 (5)
Concerns about diabetes	
Worse	0 (0)
No change	7 (17)
Improved	30 (71)
Much improved	5 (12)
Treatment satisfaction	
Worse	0 (0)
No change	3 (7)
Improved	32 (76)
Much improved	7 (17)
Would like to continue treatment	
Yes	41 (98)
No	1 (2)

### Cost-Effectiveness

Before switching to degludec, the majority of patients (*n* = 22) were taking glargine U100, which is therefore taken as an illustrative example with which to assess the potential cost impact of switching to degludec. After taking into account the average daily basal insulin dose for patients immediately before switching to degludec and the local product costs at the time of study, the average annual costs per patient for glargine U100 and degludec in patients with T1D with problematic hypoglycemia were estimated to be £376.10 and £336.51, respectively [22].

The estimated direct cost (excluding ambulance call-out and/or hospital admission) of an episode of severe hypoglycemia in patients with T1D in the greater Birmingham and surrounding area was taken as £189.20 [1]. Based on these data, it was estimated that the annual total direct cost of severe hypoglycemia per patient was £72.08 and £19.92 before and after switching to degludec. Taking into account the reduced basal dose and reduced number of severe hypoglycemic events, it is conservatively estimated that for this cohort of patients, switching to degludec saved at least £91.75 per patient per year (based on the 2016 treatment costs for degludec and glargine U100).

## Discussion

In patients with T1D and problematic hypoglycemia, switching basal insulin to degludec resulted in a significant reduction in both HbA_1c_ and total basal insulin dose without a corresponding increase in bolus dose. There were also reductions in the number of episodes of DKA and moderate and severe hypoglycemia.

Insulin therapy is required to control BG levels in patients with T1D, but is associated with hypoglycemia, the major barrier to insulin titration and optimal glycemic control [23]. Advanced age, longer diabetes duration and lower HbA_1c_ have all been associated with an increased risk of hypoglycemia in patients with diabetes [24, 25]. The phase 3b SWITCH 1 trial (NCT02034513) enrolled patients with T1D who had at least one hypoglycemia risk factor (e.g. a T1D duration of more than 15 years), and showed significantly lower rates of hypoglycemia with degludec compared with glargine U100 at equivalent HbA_1c_, with the benefit being relatively greater in magnitude than in the earlier phase 3a studies [12, 26]. We therefore anticipated that degludec would have greater benefits in patients particularly prone to hypoglycemia, and our results show that not only is the risk of hypoglycemia reduced in such patients, but there is also an improvement in glycemic control without an increase in insulin dose for the majority of patients.

Frequent episodes of hypoglycemia can lead to hypoglycemia unawareness, which is associated with altered counter-regulation and is more common in older patients with a long duration of diabetes [27]. In this prospective quality-improvement project, patients reported an improvement in recognizing symptoms of hypoglycemia, with more patients experiencing symptoms at higher BG levels [59.5–68.5 mg/dL (3.3–3.8 mmol/L)] after switching to degludec versus < 59.5 mg/dL (< 3.3 mmol/L) before switching. This is likely a result of the reduced incidence of hypoglycemia with degludec, since recurrent episodes can lead to impaired glucose counter-regulation and reduced hypoglycemia awareness [28]. In turn, impaired hypoglycemia awareness increases the risk of severe episodes between three- and fivefold [29, 30]. However, the normal hierarchy of hypoglycemia symptom recognition before cognitive dysfunction can be restored in patients if hypoglycemia is avoided [31].

In addition, we report that degludec treatment was associated with a reduced fear of hypoglycemia. Long-term effects of hypoglycemia can include behavioral changes as well as a significant anxiety or fear of future events [4]. Fear of hypoglycemia can adversely affect diabetes management and clinical outcomes through reduced adherence to medications or a reluctance of physicians to titrate insulin more aggressively [32, 33]. It is common for patients to maintain elevated BG levels because of a fear of hypoglycemia [34]; thus, reducing this fear may have contributed to the improved HbA_1c_ levels observed in this study. Furthermore, both hypoglycemia unawareness and recurrent severe hypoglycemia can lead to a fear of hypoglycemia, which in turn can reduce adherence to therapeutic decisions [35]. Thus, the clinical benefits of switching to degludec may also contribute to improved well-being and treatment adherence. Degludec was also associated with an improvement in diabetes-related QoL. Six months after starting degludec treatment, 41 of the 42 patients in this study said they would prefer to continue this treatment. Overall, degludec was found to be well tolerated, BG values were seen as more predictable compared with other basal insulins, confidence in the treatment was high, and diabetes-related concerns were reduced.

Newer, long-acting insulin analogs offer the advantage of flexibility, once-daily dosing, and less risk of hypoglycemia compared with older, cheaper human insulin analogues such as neutral protamine Hagedorn insulin [36, 37]. Basal insulin unit costs vary between the newer, long-acting basal insulins, hence comparative health economic analyses of treatments can help healthcare payers to make decisions about how to efficiently allocate resources to achieve maximum healthcare gains within the constraints of a limited budget. In our illustrative costs analysis, switching to degludec from other basal insulins was estimated to achieve substantial cost savings in this cohort of patients, despite the higher cost of degludec, due to reductions in the frequency of severe hypoglycemic events and the basal insulin dose after switching to degludec. Moreover, the estimated average costs of an ambulance call-out and a hospital admission due to hypoglycemia in the greater Birmingham and surrounding area were £242.78 and £990.96, respectively, according to recent data at the time of this study [2, 3]. Additionally, avoiding an episode of DKA in the UK had an estimated associated cost saving of between £1000.00 and £1400.00 [38]. Hence, our analysis represents a conservative estimate of cost savings with degludec, as we excluded direct costs associated with ambulance call-outs and hospital admissions for hypoglycemia in our estimation of treatment costs.

Limitations of this study include the small sample size and the variability in baseline HbA_1c_, which ranged from 5.9 to 12.2% (41–110 mmol/mol). Another limitation was the fact that patient recall and diaries were relied upon as a data source for the pre-switch 6-month interval. The study also combined data from patients on different insulin regimens and although the most commonly used insulin was glargine U100, there were 17 patients who had received insulin detemir prior to switching. The cost impact assessment was based on the glargine U100 acquisition price, which, per unit, is similar to that of insulin detemir. It is possible, however, that baseline differences between these insulins (e.g. dose discrepancies [39]) confound this analysis, which should therefore be viewed as illustrative only. On the other hand, this study shows clear benefits in terms of both improved glycemic control and tolerability from switching to degludec in a selected patient group, and it helps to redress the lack of real-world data on the effect of degludec treatment in patients with recurrent, problematic hypoglycemia or hypoglycemia unawareness.

## Conclusions

The results of this real-world study indicate that degludec may be a beneficial treatment for patients with T1D with problematic hypoglycemia. In such patients, the switch to degludec resulted in a reduced basal dose, reduced HbA_1c_ levels, improved QoL, and reduced rates of DKA and hypoglycemia. Used in appropriate targeted patients, degludec could therefore lead to significant cost savings for the NHS, despite its acquisition cost.
